# Identification of microRNA‐181 as a promising biomarker for predicting the poor survival in colorectal cancer

**DOI:** 10.1002/cam4.2520

**Published:** 2019-08-25

**Authors:** Qiliang Peng, Wenyan Yao, Chenxiao Yu, Li Zou, Yuntian Shen, Yaqun Zhu, Ming Cheng, Zhengyang Feng, Bo Xu

**Affiliations:** ^1^ Department of Radiotherapy & Oncology The Second Affiliated Hospital of Soochow University Suzhou China; ^2^ Institute of Radiotherapy & Oncology Soochow University Suzhou China; ^3^ Department of General Surgery The Second Affiliated Hospital of Soochow University Suzhou China; ^4^ Department of Oncology The Second Affiliated Hospital of Soochow University Suzhou China

**Keywords:** bioinformatics, colorectal cancer, meta‐analysis, microRNA‐181

## Abstract

**Background:**

A series of studies have investigated the vital role of microRNA‐181 (miR‐181) in the initiation and development of colorectal cancer (CRC), and demonstrated that it might be associated with the prognosis of CRC. However, inconsistent findings have hindered its clinical application.

**Methods:**

A comprehensive meta‐analysis and an integrative bioinformatics analysis were carried out for concluding current available evidence, clarifying the preliminary prognostic value and unfolding the underlying biological function of miR‐181 in CRC patients.

**Results:**

The findings revealed that elevated expression levels of miR‐181 were associated with significantly poorer overall survival rates (HR = 1.75, 95% CI: 1.26‐2.43, *P* < .05). Meanwhile, the target genes of miR‐181 were identified and enriched into several important gene ontology (GO) categories and signaling pathways including miRNAs in cancer, pathways in cancer, proteoglycans in cancer, colorectal cancer, FoxO signaling pathway, PI3K‐Akt signaling pathway, VEGF signaling pathway, HIF‐1 signaling pathway, mTOR signaling pathway, and cAMP signaling pathway, which were confirmed highly involved in the initiation and progression of CRC. In addition, the protein‐protein interaction (PPI) networks were set up by miR‐181 targets to screen hub nodes and significant modules, which were also considerably associated with the molecular pathogenesis of CRC.

**Conclusions:**

The present study demonstrated that miR‐181 could be a promising biomarker with predictive value for prognosis for CRC patients. However, future studies comprising large cohorts from multicenter are warranted to further investigate the biomarker value of miR‐181.

## Graphical abstract

A comprehensive meta‐analysis was carried out for concluding current available evidence of the biomarker role of miR‐181 in CRC. An integrative bioinformatics analysis was conducted for unfolding the underlying biological function of miR‐181 in CRC patients

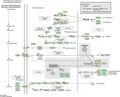

## 1 INTRODUCTION

Colorectal cancer (CRC) is one of the most common malignancies and a leading cause of cancer‐related mortality worldwide.^1^ Despite the improvement in treatment management and cancer surveillance for CRC during recent years, the overall survival rate still remains a low level due to high local recurrence rate and distal metastasis.^2^ Biomarkers that could early predict the survival outcome would be valuable for proper individual management. Thus, it is important to search convenient and effective biomarker with high prognostic value to identify high‐risk CRC patients who tend to have local recurrence or poor prognosis.

MicroRNAs (miRNAs) are a family of short, single‐stranded, and noncoding RNAs which modulate the expression of target genes and exert important roles within diverse biological courses containing cellular growth, proliferation, development, differentiation, angiogenesis, metabolism, and apoptosis.^3^, ^4^ Growing studies have identified that dysregulation of miRNAs participated in the tumorigenesis and progression of CRC.^5^ Meanwhile, miRNAs commonly exist in tissue, blood, urine, and feces, and can be easily detected. Moreover, miRNAs remain relatively stable in unfavorable physiochemical conditions including extreme variations in pH, temperature, and freeze‐thaw cycles as a hairpin‐loop structure could prevent them from enzyme or endonucleases degradation.^6^, ^7^ Considering the high stability and distinct expression profiles in human malignancies, miRNAs may be exploited as new promising molecular agents for predicting the prognosis or treatment response in CRC.^8^

As one of the most studied miRNAs, microRNA‐181 (miR‐181) family consists of miR‐181a, miR‐181b, miR‐181c, and miR‐181d.^9^ The four members of miR‐181 family are organized in clusters and have similar base sequences, which express and function synergistically. Among the evaluated members of miR‐181, miR‐181a and miR‐181b have been the most researched ones. The information gathered so far indicated that miR‐181a promoted angiogenesis in CRC through targeting SRCIN1 to promote the SRC/VEGF signaling pathway.^10^ Previous studies have presented that the inhibition of miR‐181a expression may restrain CRC cell proliferation via the signaling of PTEN/Akt pathway.^11^ Epigenetic silencing of miR‐181b may lead to tumorigenicity in CRC by targeting RASSF1A.^12^ Meanwhile, previous studies have reported that miR‐181a and miR‐181b were expressed abnormally in CRC tissue, and may be associated with unfavorable clinical outcome. However, although number of studies have been conducted, the predictive role of miR‐181 for the survival outcome of CRC individuals remains controversial because of the small study populations in single studies.

Therefore, the present study was performed to comprehensively inspect the predictive performance of miR‐181 expression level for CRC prognosis. In addition, we also explored the function of miR‐181 through an integrative bioinformatics analysis at the systems biology level.

## 2 MATERIALS AND METHODS

### 2.1 Eligibility criteria

The eligible studies included for qualitative synthesis must meet the following criteria: (a) the study assessed the biomarker role of miR‐181 for the prognosis in CRC individuals; (b) the study reported survival data including hazard ratios (HRs) with 95% confidence intervals (CIs) directly or they could be calculated by Kaplan‐Meier survival curve; (c) the study was published in English.

Studies would be excluded from this meta‐analysis if: (a) the study was not related to our research topics; (b) the study provided no sufficient information regarding the calculation of the HRs and their 95% CIs, or the Kaplan‐Meier curve could not be used to calculate the HRs and 95% CI parameters; (c) the study was published as review, letter, conference abstract, or case report.

### 2.2 Search strategy

The present study was carried out and covered in accordance with the PRISMA statement criteria.^13^ Relevant studies were identified through closely examining PubMed, Embase, Web of Science databases, and the Cochrane Library to acquire eligible studies with the following search terms: (“miRNA‐181” OR “miR‐181” OR “microRNA‐181”) AND (“colorectal” OR “colon” OR “colonic” OR “rectal” OR “rectum”) AND (“cancer” OR “tumor” OR “carcinoma” OR “neoplasm” OR “adenocarcinoma”). The latest search was conducted on 20 October 2018. The bibliographies within the included articles were manually screened to discover further potentially associated publications that may be passed by online information retrieval. Two reviewers (Peng and Yao) identified the eligible publications independently and resolved any disagreements by discussion.

### 2.3 Data extraction

Two authors (Peng and Yao) independently collected the data from all eligible studies using standardized forms. Discrepancies between two authors were resolved through discussion. A third researcher would be consulted to resolve the disagreement by collecting data from the enrolled study independently if they failed to reach a consensus. For every included study, we collected the following data elements: first author's name, publication year, country of study population, patient characteristics (median age, gender, cancer location, and TNM stage), patient numbers, sample source, miRNA detection method, follow‐up time, prognosis data including HRs as well as the corresponding 95% CIs. If the HRs and 95% CIs were not provided directly in the study, they can be acquired through reading the data of Kaplan‐Meier survival curves via a useful tool, Engauge Digitizer 4.1, based on the methods described by Tierney et al.^14^

### 2.4 Quality assessment

The methodological qualities for all included studies were evaluated on the basis of the Newcastle‐Ottawa Scale (NOS) which ranges from 0 to 9, including three parts of quality assessment: selection of study population, comparability, exposure or outcome evaluation.^15^ Studies with six or more NOS scores were deemed as high quality.

### 2.5 Data pooling and analysis

Pooled HRs with their 95% CIs from the qualified studies were combined to examine the relationships between miR‐181 and the survival outcomes of CRC patients. Heterogeneity across studies was estimated based on Cochran's Q test and Higgins's *I* ^2^ statistic. By convention, *P* < .05 or *I* ^2^ > 50% shows a significant heterogeneity among included studies.^16^ A random‐effect or fixed‐effect model was chose based on the pooled results of heterogeneity. In detail, if obvious heterogeneity was observed (*P* < .05 or *I* ^2^ > 50%), we preferred the random‐effects model; if not, we turned to the fixed‐effects model. The potential sources of heterogeneity were explored using subgroup analysis, meta‐regression analysis, and sensitivity analysis.^17^ Finally, publication bias was estimated on the basis of Egger's linear regression test and Begg's funnel plots.^18^ All analyses were performed using STATA 14.0 software. In the present study, *P* < .05 was reflected as statistically significant.

### 2.6 Identification of target genes of miR‐181

The target genes of miR‐181a and miR‐181b were collected from the powerful tool miRTarBase, which provides integrative information regarding experimentally tested and verified miRNA‐target interactions.^19^ The miRTarBase database has been most recent updated in 2018 with more adequately annotated and experimentally verified miRNA‐mRNA target pairs.

### 2.7 Functional enrichment of MiR‐181

To obtain a more in‐depth understanding of miR‐181 in the initiation and progression of CRC, an integrative bioinformatics analysis was performed. Gene ontology (GO) analysis is a very valuable method for predicting the potential functions of the target genes of miR‐181 at three different levels including the biological process (BP) level, the cellular component (CC) level as well as the molecular function (MF) level.^20^ The Kyoto Encyclopedia of Genes and Genomes (KEGG) is a knowledge database with the roles of systematic analysis of gene functions, associating genomic information with higher‐order functional data.^21^ In the current analysis, the GO function and KEGG pathway analysis were applied on the basis of the Database for Annotation, Visualization and Integrated Discovery (DAVID), which is an online bioinformatics resource designed for interpreting the function of a large number of genes or proteins.^22^ In the bioinformatics study, *P* < .05 was regarded as a statistically significant difference.

### 2.8 PPI network analysis of miR‐181

The Search Tool for the Retrieval of Interacting Genes (STRING) database is an online bioinformatics resource aimed to examine the protein‐protein interaction (PPI) information.^23^ For exploring the underlying associations across the target genes of miR‐181, all the targets of miR‐181 were submitted in STRING to obtain their PPI data and only the interactions with a combined score >0.4 were set to be the cut‐off criterion. Subsequently, the PPI networks were visualized with employing Cytoscape software.^24^ Then, the plug‐in CytoNCA of Cytoscape was used to determine the hub genes of PPI networks based on three different centrality measures, including betweenness centrality and closeness centrality and degree centrality.^25^ According to the centrality values of genes in the constructed PPI network of miR‐181 targets, the top 20 ranked nodes were chosen as the crucial genes. Meanwhile, the plug‐in Molecular Complex Detection (MCODE) of Cytoscape was employed for identifying the significant modules within the constructed network. In addition, the KEGG enrichment pathway analysis was carried out for nodes involved in the identified modules. *P* < .05 was regarded as significant.

## 3 RESULTS

### 3.1 Description and characteristics of eligible studies

**Figure 1:**
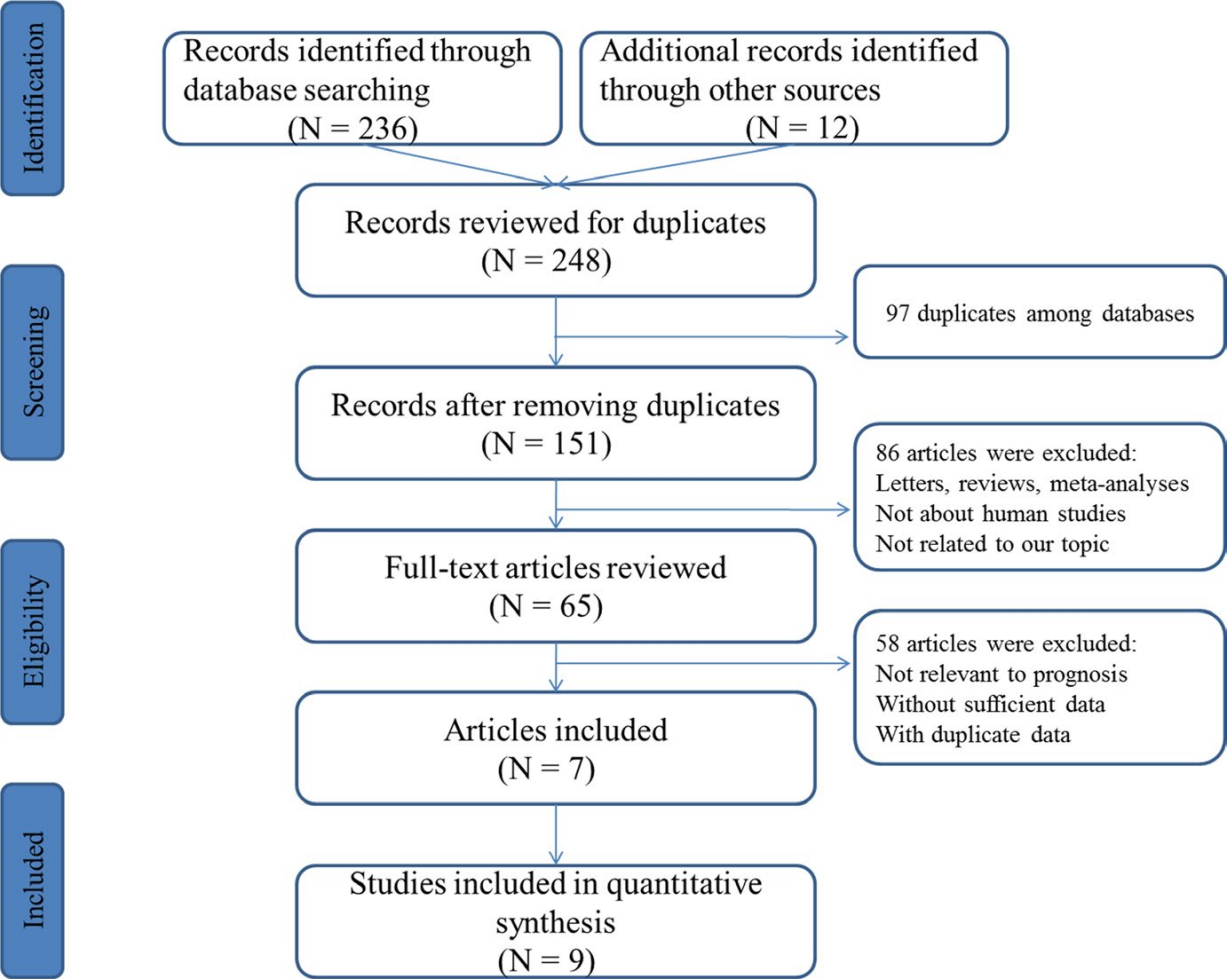
Flow diagram of the study selection process

The flowchart of detailed literature identifying process is displayed in Figure 1. A total of 248 articles were searched for initial evaluation through the selected databases. On the basis of the inclusion and exclusion criteria in the present study, seven articles with nine studies including 1017 patients were eventually identified for further evaluation, which reported the relationship between miR‐181 expression and clinical outcome data of CRC patients.^26^, ^27^, ^28^, ^29^, ^30^, ^31^, ^32^ The baseline characteristics of eligible studies are summarized in Table 1. Among all enrolled studies, five studies came from Asian, four studies from non‐Asian populations. The expressions of miR‐181 were all measured with the quantitative real‐time polymerase chain reaction (qRT‐PCR) in tissue samples. Most of the enrolled studies had adequate follow‐up time and better prognostic outcomes measurement, using pertinent statistical analysis methods. Accordingly, each eligible study had a moderate to high NOS score (ranged from 7 to 9) for assessing the prognosis of CRC.

**Table 1:** The main features of the included studies

First author	Year	Country	Ethnicity	miRNA	M/F	N	Age	TNM stage	Sample source	Methods	Endpoints	Median follow‐up time	Hazard ratio
Nakajima et al	2006	Japan	Asian	miR‐181b	25/21	46	59	I‐IV	Tissue	RT‐PCR	OS	46.7	1.22 (0.43‐3.44)
Schetter et al	2008	USA	Non‐Asians	miR‐181b	66/18	84	64.6	I‐IV	Tissue	RT‐PCR	OS	68	3.20 (1.20‐6.70)
Nishimura et al	2012	Japan	Asian	miR‐181a	98/64	162	67	I, III, IV	Tissue	RT‐PCR	OS	35.6	1.83 (1.26‐2.76)
Bovell et al	2013	UK	Non‐Asians	miR‐181b	35/71	106	65	I‐IV	Tissue	RT‐PCR	OS	228	3.55 (1.50‐8.41)
Bovell et al	2013	UK	Non‐Asians	miR‐181b	18/18	36	65	III	Tissue	RT‐PCR	OS	228	7.94 (1.60‐39.30)
Pichler et al	2013	Austria	Non‐Asians	miR‐181a	54/26	80	60	II‐IV	Tissue	RT‐PCR	OS	39	0.63 (0.37‐1.11)
Ji et al	2014	China	Asian	miR‐181a	77/60	137	59.2	I‐IV	Tissue	RT‐PCR	OS	55	1.87 (1.08‐3.25)
Ji et al	2014	China	Asian	miR‐181a	161/133	294	59.6	I‐IV	Tissue	RT‐PCR	OS	55	1.38 (1.11‐1.72)
Li et al	2016	China	Asian	miR‐181a	NA	72	NA	I‐IV	Tissue	RT‐PCR	OS	30	2.00 (1.21‐3.31)

Abbreviation: F, female; M, male; N, number; OS, overall survival.

### 3.2 Prognostic role of miR‐181 in CRC

**Figure 2:**
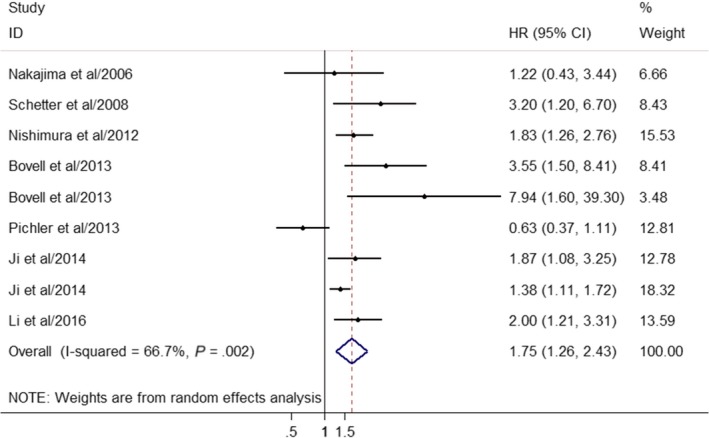
Forest plot of the correlation between miR‐181 expression level and CRC prognosis

As we found out obvious heterogeneity across the studies (*I* ^2^ = 66.7%, *P* = .002), a random‐effect model was chosen to compute the combined HR and its 95% CI. Figure 2 illustrated the forest plot of the pooled result for the association between miR‐181 and OS. Particularly, the result indicated that high expression levels of miR‐181 were demonstrated to significantly predict unfavorable OS in CRC, with the combined HR of 1.75 (95%CI: 1.26‐2.43, *P* < .05).

### 3.3 Heterogeneity analysis

**Figure 3:**
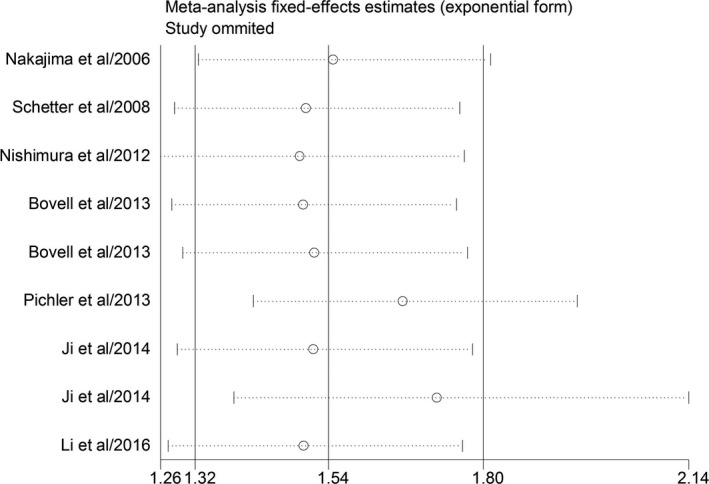
Sensitivity analysis of the meta‐analysis

As we found significant heterogeneity across the studies, we selected several common methods including subgroup analysis, meta‐regression analysis, and sensitivity analysis to seek the possible sources of heterogeneity (Table 2). We carried out subgroup analyses according to the ethnicity (Asian vs non‐Asian), miR‐181 classification (miR‐181a vs miR‐181b), and sample size (>100 vs < 100). In the Asian subgroup (five studies), high miR‐181 was significantly correlated with poor OS (HR = 1.55, 95% CI: 1.31‐1.84, *P* < .05), while for the non‐Asians, miR‐181 high expression was also related to poor OS but not significant (HR = 2.46, 95% CI: 0.78‐7.73, *P* = .124). For studies evaluating the survival outcome in different classifications of miR‐181, the results indicated that elevated miR‐181a and miR‐181b expressions both could estimate worse outcome in CRC, with the combined HR of 2.93 (95% CI: 1.57‐5.47, *P* = .03) and 1.45 (95% CI: 1.04‐2.02, *P* < .05), respectively. The sample size may alter the predictive value of miR‐181 on the OS for all involved studies. Of note, miR‐181 expression was found to be correlated with the survival outcome for sample sizes greater than 100 (HR = 1.74; 95% CI: 1.29‐3.25, *P* < .05) but not for sample sizes less than 100 subjects (HR = 1.80; 95% CI: 0.85‐3.82, *P* = .125). Afterward, the meta‐regression analysis was also carried out by the ethnicity, miR‐181 classification, and sample size to analyze the possible reasons of the heterogeneity. However, the meta‐regression analysis failed to identify the possible sources of heterogeneity. The results indicated that neither ethnicity (*P* = .719), nor miR‐181 classification (*P* = .132) or sample size (*P* = .516) might be the major source of heterogeneity. Sensitivity analysis was further carried out to examine the impact of the specific effect of individual data on the pooled HRs. The conclusion showed that there was not a single study that significantly contributed to heterogeneity after trimming individual studies, suggesting the pooled results were relatively stable (Figure 3).

**Table 2:** Results of subgroup and meta‐regression analyses

Subgroup	Studies	HR (95% CI)	*P*‐value	Heterogeneity (*I* ^2^)	*P*_heterogeneity_	Meta‐regression (*P*‐value)
miRNA classification						.132
miR‐181a	5	2.93 (1.57‐5.47)	<.05	68.7%	*P* < .05	
miR‐181b	4	1.45 (1.04‐2.02)	<.05	32.5%	*P* = .217	
Sample size						.516
Large(>100)	4	1.74 (1.29‐3.25)	<.05	47.8%	*P* < .05	
Small(<100)	5	1.80 (0.85‐3.82)	=.125	77.9%	*P* < .05	
Ethnicity						.719
Asian	5	1.55 (1.31‐1.84)	<.05	0.0%	*P* = .492	
Non‐Asian	4	2.46 (0.78‐7.73)	=.124	85.4%	*P* < .05	

### 3.4 Publication bias

**Figure 4:**
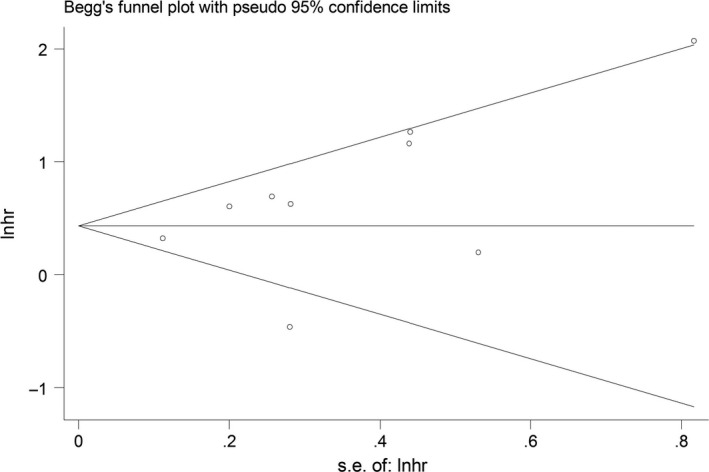
Begg's funnel plot for the assessment of publication bias in the meta‐analysis

The potential publication bias among the cohorts in miR‐181 was examined using Begg's funnel plot along with Egger's linear regression test. Although the funnel plot revealed slight publication bias (Figure 4), Egger's test demonstrated no evidence for significance in the overall results of the included studies (*P* = .215).

### 3.5 Integrative functional analysis results of miR‐181

**Figure 5:**
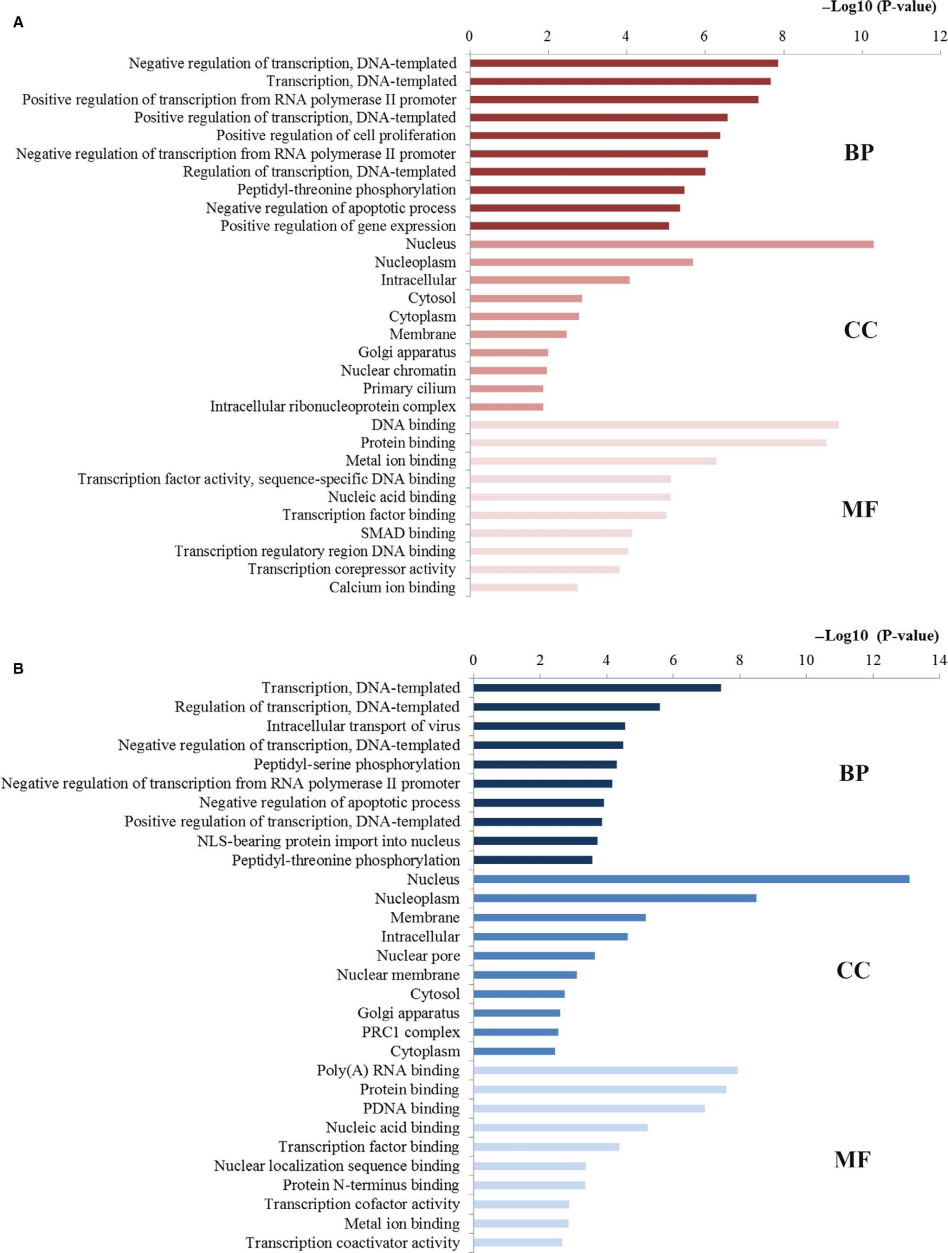
GO annotation of miR‐181 target genes. (A) Top 10 GO items for target genes of miR‐181a; (B) Top 10 GO items for target genes of miR‐106b. GO gene ontology, BP biological processes, CC cell component, MF molecular function

In order to gain a comprehensive understanding of the functions of miR‐181 in the initiation and progression of CRC, an integrated functional analyses of miR‐181 targets were carried out including GO and pathway enrichment analyses. Accordingly, a series of statistically significant GO terms were identified. Here, we mainly concentrated on the top 10 significantly enriched terms for in‐depth analyses (Figure 5). In the BP category, the targeted genes of miR‐181a were mainly linked with regulation of transcription, transcription from RNA polymerase II promoter, and cell proliferation, while the targeted genes of miR‐181b were mainly associated with the regulation processes including regulation of transcription, and apoptotic process. In the CC category, the targeted genes of miR‐181a were significantly correlated with the hallmarks of a cell containing nucleus, nucleoplasm, cytosol, and cytoplasm, while the targeted genes of miR‐181b were involved in basic cell components such as nucleus, nucleoplasm, membrane, and nuclear pore. In the MF category, the targeted genes of miR‐181a were related to the binding function including binding of DNA, protein, nucleic acid, and transcription factor, while the targeted genes of miR‐181b also converged on the binding function such as poly(A) RNA, protein, DNA, nucleic acid, and transcription factor.

**Figure 6:**
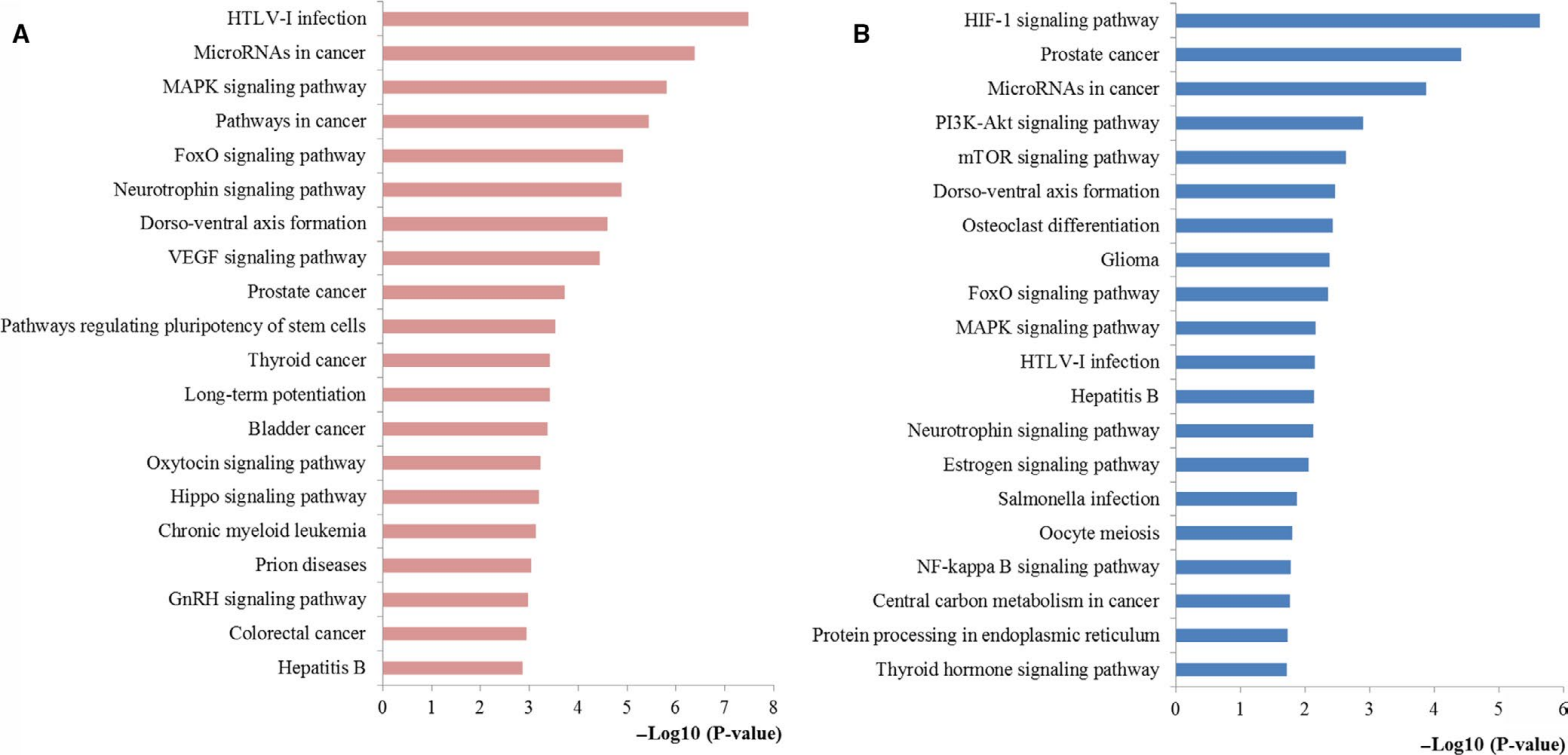
Pathway enrichment results for miR‐181 target genes. (A) Top 20 pathways enriched by target genes of miR‐181a; (B) Top 20 pathways enriched by target genes of miR‐181b

**Figure 7:**
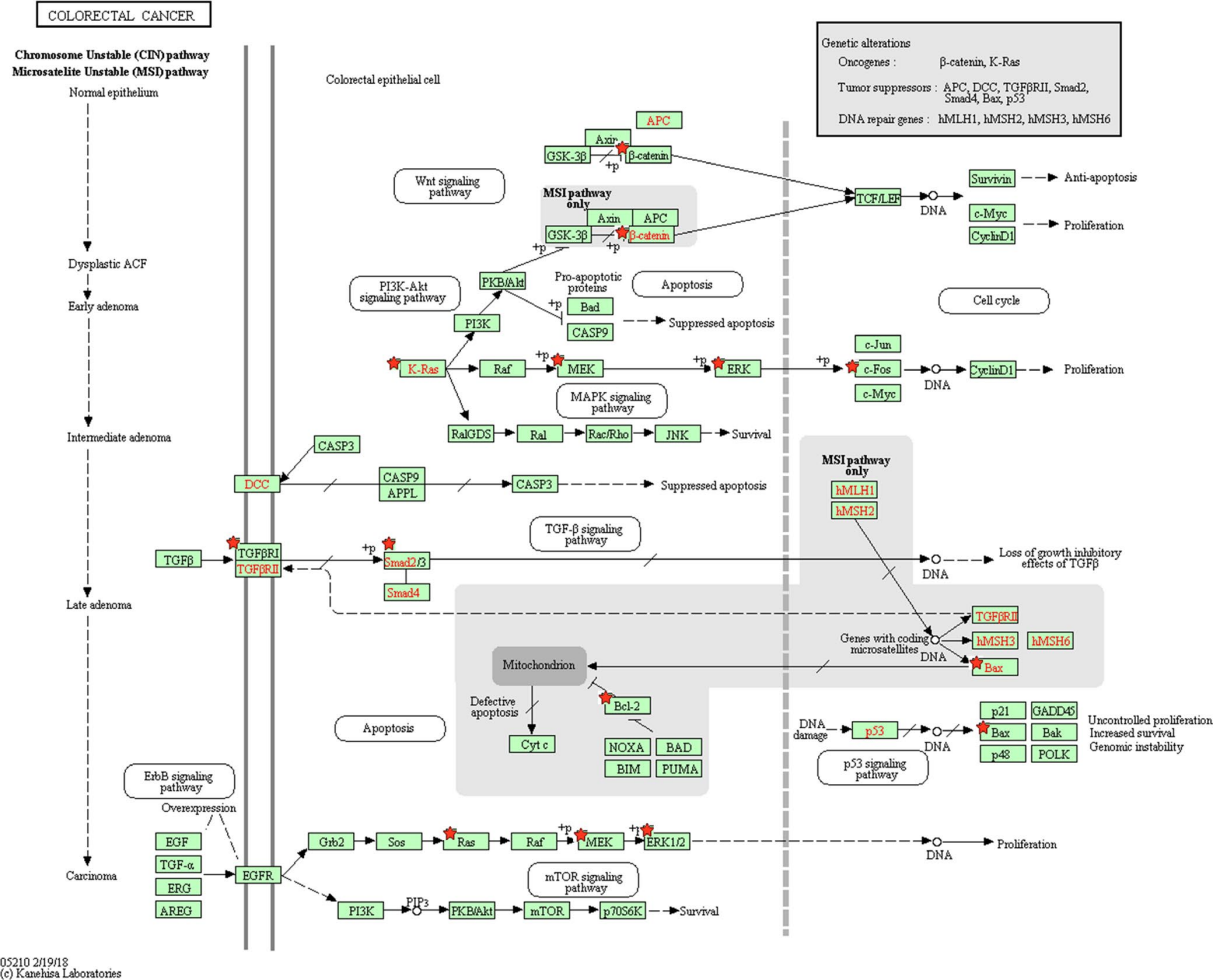
The colorectal cancer pathway enriched in KEGG. Objects with pentagrams are acting locus by mapped genes

The significantly enriched KEGG pathway terms were screened. Here, the top 20 significant terms were mainly selected for further literature exploration. As shown in Figure 6, the most meaningful KEGG terms for miR‐181a targets were miRNAs in cancer, MAPK, pathways in cancer, FoxO, VEGF, and colorectal cancer while the most significant KEGG terms for miR‐181b targets were HIF‐1, miRNAs in cancer, PI3K‐Akt, mTOR, FoxO, MAPK, and central carbon metabolism in cancer. As shown in Figure 7, the targets enriched in the colorectal cancer pathway had also close relations with PI3K‐Akt, Wnt, cell cycle, p53, and TGF‐βsignaling.

### 3.6 PPI network construction and identification of key nodes

**Figure 8:**
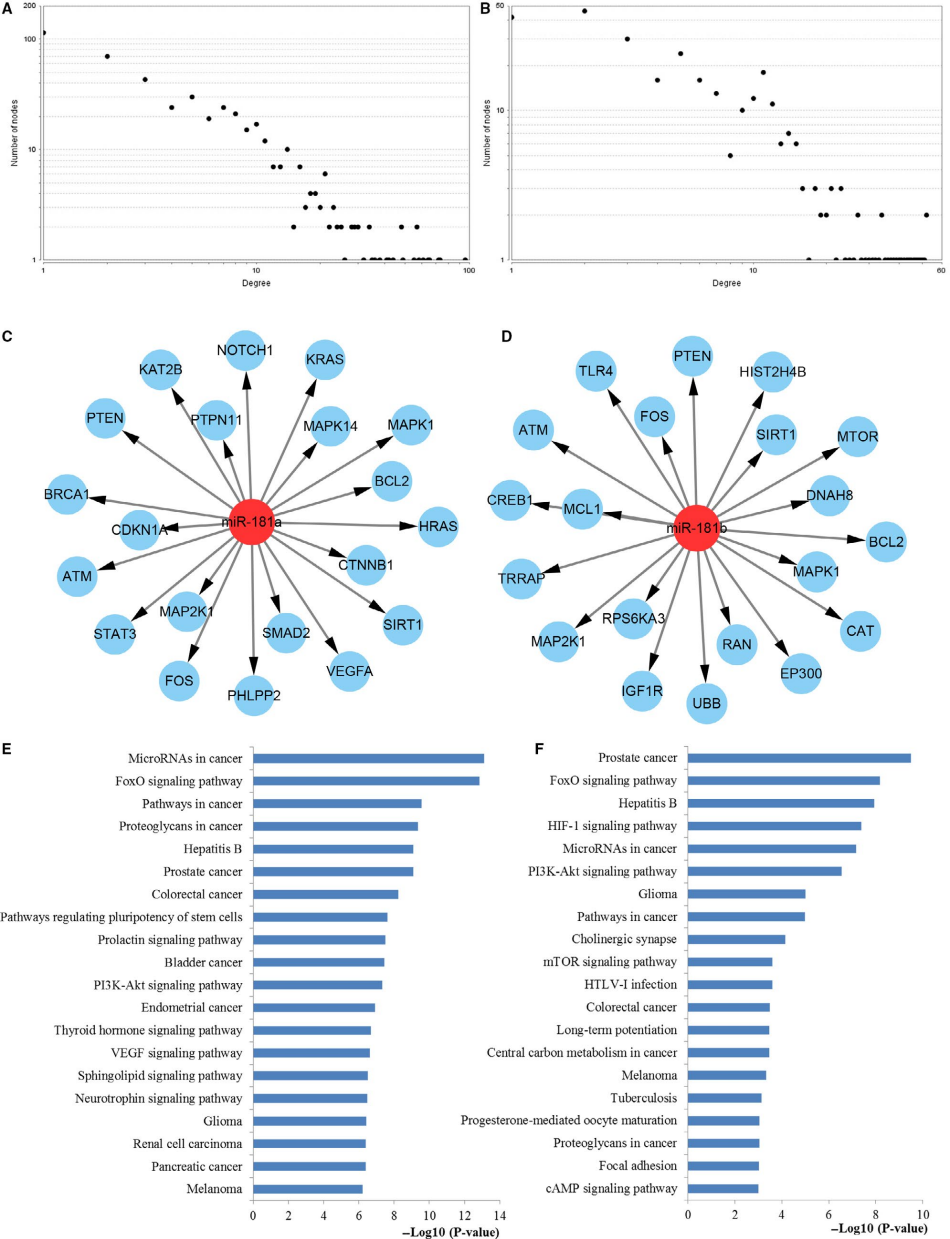
PPI network construction results. (A) Degree distributions of nodes for the network set up with miR‐181a targets; (B) Degree distributions of nodes for the network constructed with miR‐181b targets; (C) Hub genes of the network for miR‐181a targets; (D) Hub genes of the network for miR‐181b targets; € Pathway enrichment results for the selected hub genes of miR‐181a targets network; (F) Pathway enrichment results for the selected hub genes of miR‐181b targets network

To further understand the pathogenic mechanisms of miR‐181 in CRC occurrence and development, we investigated the interactions among the target genes of miR‐181a and miR‐181b. The PPI information was retrieved from STRING and integrated to construct the networks of miR‐181a and miR‐181b targets, respectively. As a result, a network of statistical significance containing 480 proteins was obtained with the set of 635 target genes of miR‐181a while a statistically significant network constructed by 294 proteins was screened with the set of 442 target genes of miR‐181b. The degree distributions for the network proteins were presented in Figure 8A,B. Then, the crucial hub genes of the PPI networks set up with the genes regulated by miR‐181a and miR‐181b were identified based on three different centrality measures containing betweenness centrality, closeness centrality, and degree centrality. The top 20 hub nodes targeted by miR‐181a and miR‐181b were illustrated in Figure 8C,D, respectively.

KEGG pathway enrichment analyses were carried out to evaluate the characteristics of the key hub nodes (Figure 8E,F). The results of the enrichment analysis indicated that the miR‐181a targets were enriched for miRNAs in cancer, FoxO, pathways in cancer, proteoglycans in cancer, colorectal cancer, PI3K‐Akt, and VEGF pathway while the miR‐181b targets were enriched for FoxO, HIF‐1, miRNAs in cancer, PI3K‐Akt, pathways in cancer, mTOR, colorectal cancer, central carbon metabolism in cancer, Proteoglycans in cancer, and cAMP pathway.

### 3.7 Module screening from the PPI network

**Figure 9:**
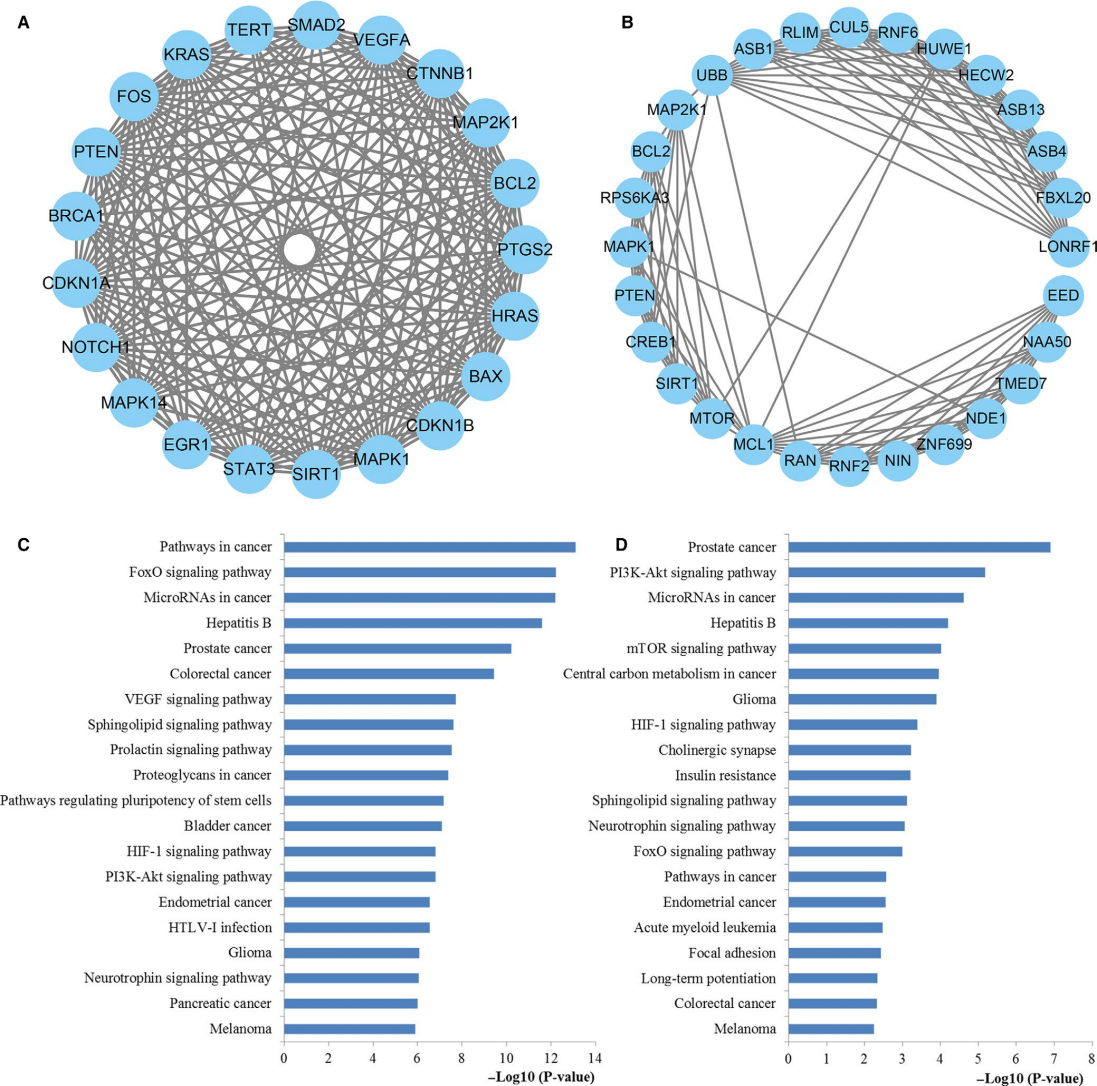
Module analysis results of the PPI network. (A) The significant module in the PPI network for miR‐181a targets; (B) The significant module in the PPI network for miR‐181b targets; (C) Pathways enriched by all the nodes involved in the identified module for miR‐181a; (D) Pathways enriched by all the nodes involved in the identified module for miR‐181b

As plotted in Figure 9A,B, the most significant modules in the PPI networks constructed by miR‐181a and miR‐181b targets were identified. Then, the genes took part in the screened modules were enriched by KEGG functional analysis (Figure 9C,D). According to the analysis, the genes involved in the significant modules of the network constructed by miR‐181a targets were closely associated with a series of significant pathways containing pathways in cancer, FoxO, miRNAs in cancer, colorectal cancer, VEGF, proteoglycans in cancer, HIF‐1, and PI3K‐Akt. Meanwhile, the genes participated in the screened modules of miR‐181b targets network were highly related to some vital pathways including PI3K‐Akt, miRNAs in cancer, mTOR, central carbon metabolism in cancer, HIF‐1, FoxO, pathways in cancer, and colorectal cancer pathway.

## 4 DISCUSSION

With the understanding and knowledge of miRNAs function in different kinds of cancer occurrence, progression, and metastasis, miRNAs attract great attention to act as preferable biomarkers for cancer diagnosis, prognosis, and therapy. Among the most representative miRNAs, miR‐181 family have been demonstrated to exert a considerable part in the development and progression of CRC, and may be promising diagnostic and prognostic biomarkers for patients with CRC. Despite increasing evidences suggested that high miR‐181 expression was significantly correlated with undesirable survival outcome on CRC, their conclusions remain controversial and most of these studies possessed limited study populations. Then, we systematically reviewed the published studies and performed this study to estimate the prognostic significance of miR‐181 expression for CRC patients. Additionally, an integrative functional analysis was performed to promote the understanding of underlying mechanisms of miR‐181 in CRC.

In this study, the results showed that increased expression of miR‐181 could be used as a prognostic biomarker, as high expression of miR‐181 correlated with poorer OS in CRC (HR: 1.75, 95%CI: 1.26‐2.43, *P* < .05). According to the pooled results, sample size seemed to have a significant role on the predictive role of miR‐181 expression for CRC patient prognosis as studies with larger sample sizes were associated with more obvious poor outcomes, which indicated that more large‐scale prospective researches are warranted to further evaluate the predictive role of miR‐181 for the prognosis of CRC. In particular, the predictive efficacy for miR‐181 was more significant in Asians than in non‐Asians, revealing that miR‐181 may be a more effective predictor of poor survival in individuals suffered with CRC for Asian populations. In addition, both miR‐181a and miR‐181b could serve as promising biomarkers for predicting the survival outcome of CRC patients. Sensitivity analysis demonstrated that our meta‐analysis was highly robust. Meanwhile, no obvious publication bias was observed in our results.

We supposed that if miR‐181 affects the prognosis of CRC patients, the target proteins adjusted by miR‐181 may also participate in the occurrence and development of CRC. Therefore, an integrated functional analysis was performed to explore the function of miR‐181 family at the systems biology level. According to the enrichment analysis, although miR‐181a and miR‐181b located on different areas of chromosome, the target genes of them were enriched into relevant functional modules, pathways, or networks and then become more concerted, revealing that miR‐181a and miR‐181b may have a synergistic effect in the initiation, progression, and pathogenesis of CRC. In particular, most GO terms enriched by the targets of miR‐181a and miR‐181b both considerately concentrated on the regulation processes for the BP column, closely associated with vital cellular structure for the CC column, and particularly linked with the binding function for the MF column, which illustrated the underlying biomarker performance of miR‐181 in the establishment and progression of CRC. It was indicated from the results that the genes targeted by miR‐181a and miR‐181b were involved in similar pathways closely related to CRC initiation and development, such as miRNAs in cancer, MAPK, pathways in cancer, FoxO, VEGF, colorectal cancer, HIF‐1, PI3K‐Akt, mTOR, and central carbon metabolism in cancer. The miRNAs in cancer pathway directly reflected the associations across the target genes and miRNAs in cancer. In addition, the pathways in cancer and colorectal cancer pathway convinced that miR‐181 was indeed associated with the initiation and progression of CRC. The MAPK signaling pathway has been verified to be fundamental for regulating a series of cellular behaviors associated with tumor development, containing proliferation, invasion, apoptosis, and immune escape.^33^ Emerging evidence has supported that activation of MAPK signaling is significantly correlated with the pathogenesis, progression, and oncogenic activities of human CRC.^34^ There is growing evidence that FOXO signaling pathway plays an significant role in accommodating numerous biological behaviors from progression, cell signaling, and carcinogenesis to cellular metabolism and the abnormal activation of FOXO signaling pathway may bring about the physiological alterations toward carcinogenesis.^35^ Studies have convinced the roles of VEGF signaling pathway in the vascularization and growth of primary tumors and in the formation of metastases.^36^ Previous evidence has indicated that miR‐181a provides a central node to promote angiogenesis in CRC by targeting SRCIN1 to promote the VEGF signaling pathway.^10^ HIF‐1, an important signaling pathway, is highly involved in regulating cancer dormancy and metabolism, increasing stemness activity, and resulting in cancer initiation and progression.^37^ PI3K‐AKT signaling pathway has been identified as one of the important dysregulated pathways in different tumor types, including CRC. It is well established that PI3K‐AKT signaling leads to CRC tumorigenesis through reducing apoptosis, stimulating cell growth, and increasing proliferation and metastases.^38^ The well‐studied mTOR signaling pathway, which is frequently activated in various cancer types including CRC, plays crucial and complex roles in controlling cell growth, survival, and metastasis.^39^ Central carbon metabolism has been studied for years for monitoring cancer progression and therapy response due to the increased need for cellular biomass in cancer.^40^ The imbalance of carbon metabolism regulation will lead to carcinogenesis.^41^ The functional enrichment results not only demonstrated the robustness of our study but explained why miR‐181 could possess such excellent biomarker features in predicting the survival outcome of CRC to some extent.

On the basis of an analysis of the PPI network, a series of core genes of miR‐181a and miR‐181b was identified. It could be revealed from the pathway enrichment analysis that the hub genes were associated with miRNAs in cancer, FoxO, pathways in cancer, proteoglycans in cancer, colorectal cancer, PI3K‐Akt, VEGF, HIF‐1, mTOR, central carbon metabolism in cancer, and cAMP signaling pathway. Most of these pathways have been demonstrated related to CRC initiation and progression through literature exploration. In addition to the pathways mentioned above, other parts have to describe were the proteoglycans in cancer and cAMP signaling pathway. In recent years, accumulating new evidence supports the concept that proteoglycans in cancer pathway played important roles in extracellular matrix structural organization and cell signaling causing the control of a multitude of normal and pathological processes.^42^ Emerging studies suggest that aberrant activation of proteoglycans in cancer pathway may contribute to tumorigenesis, cancer progression, and treatment resistance.^43^ The cAMP signaling pathway is one of the most ancient signaling able to modulate substantial biological behaviors containing cellular growth and differentiation, gene expression, as well as neuronal function.^44^ It is now appreciated that aberrant activation of cAMP signaling may activate multiple effector proteins at distinct intracellular areas and then contribute to tumorigenesis. The PPI analysis further revealed the function of miR‐181 in the tumorigenesis and progression of CRC. The identified hub genes may provide potential therapeutic targets for improving the chemotherapy effect of CRC.

Module analysis identified the most significant modules of the constructed networks of miR‐181a and miR‐181b targets. The pathway enrichment analysis demonstrated that the nodes were significantly correlated with pathways in cancer, FoxO, miRNAs in cancer, colorectal cancer, VEGF, proteoglycans in cancer, HIF‐1, PI3K‐Akt, mTOR, and central carbon metabolism in cancer. Most of the enriched pathways were involved in CRC occurrence and development according to PubMed literature reports mentioned above. Additionally, these pathways were repeatedly mentioned in KEGG pathway analysis enriched by all the miR‐181a and miR‐181b targets, key hub targets, and network modules, revealing their vital roles in the initiation and progression of CRC, which may provide new ideas for the therapeutic targets of CRC. These pathways should be given top priority for further study.

CRC is a highly complex disease driven by extensive genetic and epigenetic alterations. Single miR‐181 is not sufficient to serve as a perfect tumor biomarker for CRC. According to the previous studies, some CRC biomarkers, including miR‐29, miR‐92a, miR‐106, and other miRNAs, also took part in some important pathways that were involved by miR‐181 such as miRNAs in cancer, FoxO signaling, pathways in cancer, proteoglycans in cancer, colorectal cancer signaling, and PI3K‐Akt signaling.^45^, ^46^, ^47^ It may suggest that these cellular signaling that can lead to poor prognosis of CRC may be modulated by a series of miRNA biomarkers. Moreover, previous evidence has demonstrated that miR‐181 along with five miRNA biomarkers (miR‐21, let‐7g, miR‐31, miR‐92a, and miR‐203) had high sensitivity and specificity for distinguishing the CRC samples from normal controls. In addition, the six‐miRNA signature including miR‐181 was also correlated with CRC progression.^48^ In another study, miR‐181 was identified as an effective member of 16‐miRNA signature for classifying colon cancer stage II and III patients into groups with low and high risk for recurrence.^49^ In all, miR‐181 and other types of miRNAs have a synergistic effect in modulating cellular signaling that can lead to poor prognosis.

Our study has several inspiring points. Firstly, miR‐181 was proved as a valuable prognostic biomarker for CRC patients as miR‐181 overexpression was closely related to unfavorable clinical outcome. Secondly, our results indicated that high expression level of miR‐181 could predict poor OS both in Asians and non‐Asians with CRC. It followed that miR‐181 may be applied to different genetic background. Thirdly, compared to conventional meta‐analysis, our study not only quantitatively analyzed the prognostic role of miR‐181 in predicting the overall survival of CRC patients, but also explored its function through an integrative bioinformatics analysis. What's more, we explained the potential mechanisms for miR‐181 involved in the occurrence and progression of CRC. Finally, the current study emphasized the potential role of miR‐181 in acting as a hopeful therapeutic target for CRC patients.

However, there were also limitations of our study that we should attend. First, the studies enrolled for the present qualitative synthesis were mostly observational researches, which inherently contained greater potential for confounding than perspective researches. Then, the numbers of the involved studies were relatively limited, and the conclusion of our analysis results should be interpreted with caution. Therefore, more relevant researches are required in the future. Moreover, we cannot deal with the patients' survival data in a consistent form as the data were not collected from individual patient data. In the end, only English researches were included in our study, which could cause the existence of potential publication bias as studies published in English would likely include increased proportions of positive results.

## 5 CONCLUSIONS

To sum up, it was preliminarily concluded that elevated expression of miR‐181 is potentially correlated with the worse OS in CRC patients, and may act for a promising biomarker for prognosis. An integrative bioinformatics analysis was taken to explore the function of miR‐181 at the systems biology level, further explained the reason why miR‐181 family could be used as a biomarker for CRC. However, more prospective researches with large sample size and biological experiments must be conducted for focusing on further confirming the role of miR‐181 in CRC as the sample size of this study is still limited for making meaningful conclusions.

## CONFLICT OF INTEREST

The authors declare they have no competing interest.

## ETHICS APPROVAL AND CONSENT TO PARTICIPATE

Not applicable.

## Data Availability

The data supporting the conclusions of this article are within the article.
